# Intraoperative Challenge: Managing Venous Air Embolism During Sitting Craniotomy

**DOI:** 10.7759/cureus.61484

**Published:** 2024-06-01

**Authors:** Angan Ghosh, Sanjot Ninave

**Affiliations:** 1 Anaesthesia, Jawaharlal Nehru Medical College, Datta Meghe Institute of Higher Education and Research, Wardha, IND

**Keywords:** patient safety, intraoperative management, complications, neurosurgery, craniotomy, venous air embolism

## Abstract

Venous air embolism (VAE) represents a rare yet potentially life-threatening complication encountered during neurosurgical procedures, particularly craniotomy. Here, we present a case of a 30-year-old male undergoing excision of a cerebellar abscess who developed VAE midway through the procedure. Immediate recognition and intervention were paramount in managing the embolism effectively, ensuring a favorable surgical outcome. Vigilant monitoring, prompt cessation of the procedure, and implementation of preventive measures such as oxygen therapy and venous air aspiration were pivotal in mitigating the embolism's effects. This study underscores the critical importance of intraoperative vigilance, preparedness, and multidisciplinary teamwork in addressing rare but potentially catastrophic complications during neurosurgical interventions.

## Introduction

Venous air embolism (VAE) is a rare but potentially fatal complication that can occur during various surgical procedures, including neurosurgery, such as craniotomy [1]. VAE arises from the inadvertent introduction of air into the venous system, leading to obstructive effects on pulmonary circulation and systemic embolization, resulting in cardiovascular compromise and neurological sequelae [2]. While the reported incidence of VAE during neurosurgical procedures is low, ranging from 0.13% to 0.61%, its occurrence necessitates immediate recognition and intervention to prevent adverse outcomes [3]. The pathophysiology of VAE involves the formation of air bubbles within venous channels, which can occur due to several mechanisms, including direct communication between a surgical field and venous structures, positive pressure ventilation, or venous catheterization [4]. In neurosurgery, procedures involving the manipulation of venous sinuses, such as craniotomy, pose an increased risk of VAE due to the proximity of the surgical site to major venous structures.

Clinical manifestations of VAE can vary depending on the volume and rate of air entry, ranging from mild symptoms such as tachycardia, hypotension, and dyspnea to severe manifestations including cardiac arrest, pulmonary edema, and neurological deficits. Diagnosis is often based on clinical suspicion supported by intraoperative monitoring modalities such as transesophageal echocardiography, end-tidal CO_2_ monitoring, and arterial blood gas analysis [5]. Management of VAE during craniotomy involves a multi-faceted approach aimed at preventing further air entry, reducing air volume, and optimizing tissue oxygenation. Immediate measures include halting the surgical procedure, positioning the patient to prevent air entry into the heart, administering 100% oxygen to accelerate nitrogen washout, and aspirating air from central venous catheters [6]. Despite advancements in intraoperative monitoring and surgical techniques, VAE remains a formidable challenge in neurosurgery, emphasizing the importance of vigilance, preparedness, and prompt intervention to ensure optimal patient outcomes.

## Case presentation

A 30-year-old male presented to the outpatient department of the tertiary care hospital in Wardha district with a chief complaint of difficulty in walking persisting for the past 30 days, accompanied by episodes of giddiness, fever, and vomiting over the last 15 days. Upon taking the patient's medical history, he also complained of lightheadedness and dizziness on standing after lying down, suggestive of postural hypotension.

**Figure 1:**
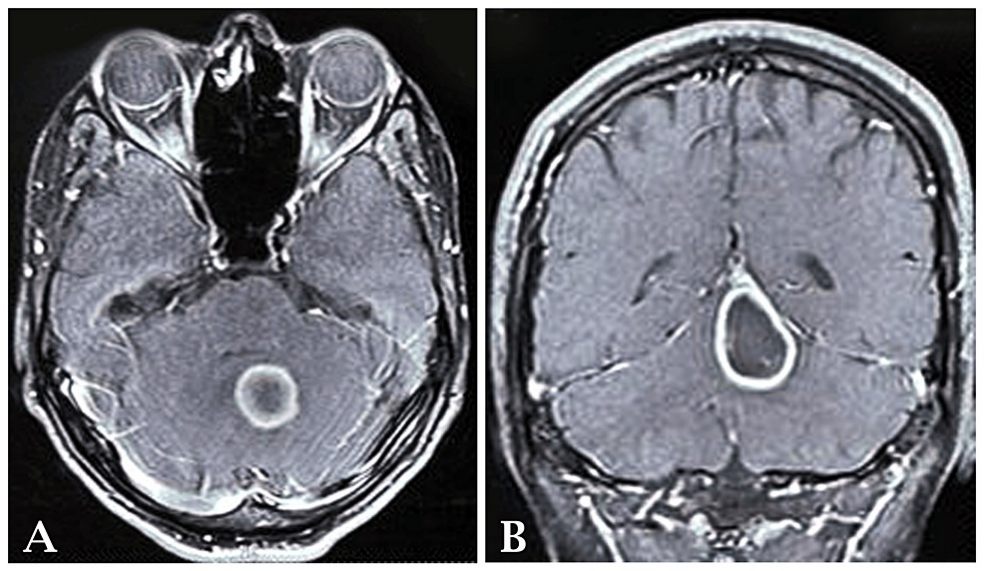
(A and B) The images show the presence of rounded to oval-shaped ring-enhancing lesions involving the left cerebellar vermis, displaying hypointense T1W1 and hyperintense T2W1 and FLAIR signals, with mild restricted diffusion on diffusion-weighted imaging (DWI), and perilesional vasogenic edema exerting mass effect on the fourth ventricle, indicating a likely abscess or neoplastic lesion. FLAIR: fluid-attenuated inversion recovery

Although routine investigations were within normal limits, 2D echocardiography yielded a left ventricular ejection fraction of 53-54% with right ventricular systolic pressure (RVSP) of 15 mmHg plus right atrial pressure (RAP). However, upon performing computed tomography (CT) scans of the brain, both plain and post-contrast axial images revealed a well-defined, round, post-contrast rim-enhancing lesion predominantly hypodense in the cerebellar region, measuring approximately 20x20 mm in size, with surrounding edema suggestive of early abscess formation. Magnetic resonance imaging (MRI) with contrast further confirmed the presence of rounded to oval-shaped ring-enhancing lesions involving the left cerebellar vermis, displaying hypointense T1W1 and hyperintense T2W1 and fluid-attenuated inversion recovery (FLAIR) signals, with mild restricted diffusion on diffusion-weighted imaging (DWI), and perilesional vasogenic edema exerting mass effect on the fourth ventricle, indicating a likely abscess or neoplastic lesion. Based on these clinical and radiological findings, a diagnosis of brain abscess was made by the neurosurgeon Figures 1A, 1B.

**Figure 2:**
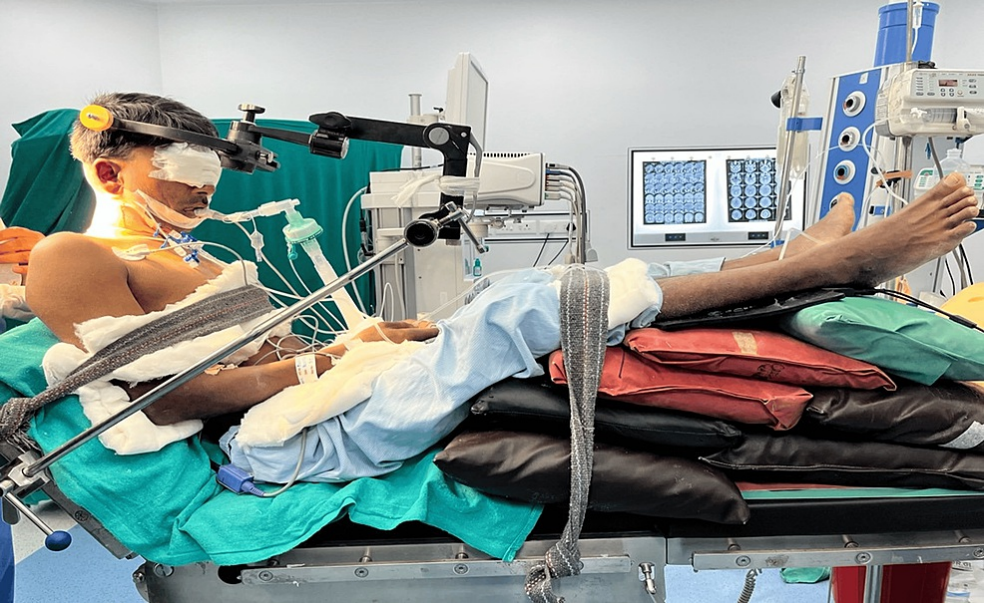
Shows intraoperative setup during sitting craniotomy

Following diagnosis, immediate treatment was initiated, involving intravenous antibiotic therapy, with plans for surgical intervention outlined after obtaining written consent from both the patient and his relative. Subsequently, the patient underwent excision of the abscess via sitting craniotomy in the operating theatre under general anesthesia. After confirming the nil per oral status of the patient, the patient was connected to the multiparameter monitor, and baseline vitals were recorded. A 20 gauge IV cannula was secured in the left upper limb and an 18 gauge IV cannula in the left lower limb. The patient was preoxygenated with 100% oxygen for four minutes with an anatomical face mask and premedicated with an injection of lignocaine hydrochloride 2% (1 mg/kg) and an injection of fentanyl (2 mcg/kg). Induction was done with an injection of sodium thiopentone (5 mg/kg) and inhalational agent sevoflurane. Muscle relaxation was achieved with the injection of vecuronium (0.12 mg/kg). Intubation was done with a 7.5 mm cuffed endotracheal tube, confirmed with capnography and five-point auscultation. The arterial line was secured at the right radial artery for invasive arterial blood pressure monitoring, followed by a triple lumen central line insertion at the right internal jugular vein. The sitting position was given incrementally over 10-15 minutes until the toes were at the vertex level, and adequate padding of the pressure points was ensured (Figure 2). Injection levetiracetam was given to prevent intraoperative convulsions and injection of mannitol (prevent brain edema and for intraoperative brain relaxation. Maintenance of anesthesia was done with the inhalational agent sevoflurane, a mixture of oxygen and air, and an injection of vecuronium (0.02 mg/kg). Analgesia was achieved with hourly doses of injection fentanyl (1 mcg/kg). Oxygen saturation was maintained at 100%, and capnography was between 31 mmHg and 36 mmHg.

Hemodynamic stability was ensured throughout the procedure, with appropriate fluid resuscitation, including 1 unit of packed red cells (PRC) transfused and 5 L of Ringer lactate and normal saline alternately. The surgical duration was approximately 5 h, during which a urine output of 1200 mL and estimated blood loss of 580 mL was noted.

However, midway through the procedure, the patient’s EtCO_2_ level suddenly dropped from 32 mmHg to 16 mmHg, prompting suspicion of a venous air embolism. The surgical team promptly halted the procedure, covering the surgical site with saline-soaked mops and giving Trendelenburg position. A 100% oxygen was administered to maximize oxygenation. Aspiration was performed through the central line to alleviate the air embolism while maintaining arterial blood pressure with appropriate fluid therapy and vasopressor support [7]. These immediate interventions effectively managed the venous air embolism, ensuring patient safety and optimizing the outcome of the surgical procedure.

## Discussion

Venous air embolism (VAE) during neurosurgical procedures, such as craniotomy, presents a significant challenge due to its potential for severe complications. In this case, the prompt recognition and management of VAE were crucial in ensuring a favorable outcome for the patient. VAE occurs when air enters the venous circulation, obstructing pulmonary blood flow and subsequent hemodynamic compromise. In craniotomy, VAE can occur due to the disruption of venous sinuses or dural venous channels, allowing air entry into the bloodstream. The patient's supine position during surgery further facilitates the ascent of air to the heart, increasing the risk of embolism [3]. Several factors contribute to the development of VAE during craniotomy, including head-up positioning, a pressure gradient favoring air entry, and high-speed drills or suction devices, which can create negative pressure in the surgical field [8]. Additionally, large surgical cavities, as seen in tumor resection or abscess drainage cases, provide ample space for air accumulation [9].

Clinical manifestations of VAE vary depending on the air-embolized volume and the entry rate. Mild cases may present with nonspecific symptoms such as tachycardia, hypotension, and dyspnea, while severe cases can result in cardiovascular collapse, arrhythmias, and neurological deficits [4]. Management of VAE requires prompt recognition and implementation of preventive measures and treatment strategies. Intraoperatively, measures such as maintaining a meticulous surgical field, minimizing the use of high-speed instruments, and utilizing head-down positioning can reduce the risk of VAE [10]. In the event of suspected or confirmed VAE, the surgical team should immediately halt the procedure, cover the surgical site with saline-soaked dressings, and administer 100% oxygen to promote the elimination of nitrogen from the embolus [11]. Jugular venous compression can prevent further air entry into the heart, while aspiration through central lines can remove air from the circulation [12].

## Conclusions

In conclusion, the successful management of VAE during craniotomy underscores the critical importance of prompt recognition and immediate intervention in intraoperative complications. This study vividly illustrates the necessity for surgical teams to maintain a high level of vigilance and preparedness and the vital role of effective communication and collaboration among team members. By promptly halting the procedure upon suspicion of VAE, implementing preventive measures such as covering the surgical site with saline-soaked dressings, and initiating appropriate treatment strategies, including oxygen therapy and venous air aspiration, the surgical team effectively safeguarded the patient's well-being. It optimized the outcome of the neurosurgical intervention. Furthermore, this study serves as a reminder of the ongoing need for healthcare professionals to remain abreast of current literature and procedural protocols and engage in continued education and training to enhance their ability to respond swiftly and effectively to intraoperative emergencies. Overall, the successful resolution of VAE, in this study, underscores the multidisciplinary teamwork, clinical expertise, and unwavering commitment to patient safety characterizing modern neurosurgical practice.
